# Supplementary material for: Chromothripsis during telomere crisis is independent of NHEJ, and consistent with a replicative origin
Source: Genome Res. 2019 May;29(5):737–49. doi: 10.1101/gr.240705.118 (PMC6499312; doi:10.1101/gr.240705.118)
Supplement: Supplemental Material [file supp_gr.240705.118_Supplemental_file_1.zip › contigs/annotated_contigs/DB48/contig.2.DB48_length_521_mean_cov_7.19769673704.docx]

**DB48_length_521_mean_cov_7.19769673704**

```
CCATGCCTGCATGGCAATGGGTCCAAACGAGGGTGCCAGAGGGGCCAGTTGCAGGCCCTGAAAGGGTCGGAGGAGGATGCCAGGGTCGT
 >chr17:76482707-76482884 + E=7e-95
GGGGGCTGTGCCCTCTCTGGGGTTGCTAGCTATGGGGGTGCTGTAGGTCTAGAACAGTTGGAAGCCATTCTTGCTACCACACGG|GAAG
                                                                                     >ch
|AAAAAAAATGAGTCTCTGTCTAAAGAGGCACGTCTGCCGGTCAGCGCAGGGCAGGCTGGCTGTGTGTGTTAACAGGAGCTTCCTGCTT
r17:76483284-76483632 - E=6e-198
AGGCCCTGGGCATCCTGGAAATGATGCACGTGCTGAGCTCACTGGCACTCGAGGGGGCCAGGCACTAGTGATGCTTTCAGCAGATGCTG

ATTTGGCAACACTTACCCTGGACCAGGCCCCCTTCTCTGAGCTTTAAAAATATCAGCCCATGTAGTCCTTGGAAATGAGGGTCATGGAA

ACATCACCCCTGTTTACAGATGAGAAACCAGGGACTCAGGCATGTGCAGTAACCTGCCTAAAGCAGCAGCTGGCCATG
```
